# Sigmoid colon perforation in a SARS-CoV-2 positive neonate: a uniqueness report and a brief review

**DOI:** 10.1186/s12887-022-03392-1

**Published:** 2022-06-03

**Authors:** Zahra Jamali, Reza Sinaei, Elahe Raeisi Estabragh, Ahmad Ahangaran

**Affiliations:** 1 Department of Pediatrics, School of Medicine, Kerman University of Medical Sciences, Kerman, Iran; 2 Endocrinology and Metabolism Research Center, Institute of Basic and Clinical Physiology Sciences, Kerman University of Medical Sciences, Kerman, Iran; 3 Clinical Research Development Unit, Afzalipour Hospital, Kerman University of Medical Sciences, Kerman, Iran; 4 Department of Surgery, School of Medicine, Kerman University of Medical Sciences, Kerman, Iran

**Keywords:** SARS-CoV-2, COVID-19, Neonate, Newborn, Intestinal perforation, Sigmoid perforation, Colonic perforation, NEC

## Abstract

**Background:**

Despite the relative prevalence of small bowel and proximal colon perforation in the neonatal period, recto-sigmoid perforation is extremely rare. Full-term neonates experience intestinal perforation less frequently than premature infants. Here we report a neonate with sigmoid perforation and simultaneous Severe Acute Respiratory Syndrome Coronavirus-2 (SARS-CoV-2) infection.

**Case presentation:**

A 2550 g female neonate born at 38-weeks’ gestation from a coronavirus disease-2019 (COVID-19) infected mother by cesarean section. Despite a good Apgar score in the first and fifth minutes, she was admitted to the neonatal intensive care unit with grunting and mild respiratory distress. She underwent antibiotics and oxygen by head box resulting in an Oxygen Saturation rate of 94%. The patients’ respiratory distress decreased during the second day, resulting once breastfeeding without tolerance. While she passed meconium in the first 2 days, she developed abdominal distention on day 3. The nasopharyngeal SARS-CoV-2 real-time polymerase chain reaction (RT-PCR) was performed with positive results. Surgical consultation was requested and a thoraco-abdominal X-Ray was performed at this stage, which suspected to be a gastrointestinal perforation.

Due to clinical deterioration and persistent abdominal distention, a contrast study was performed with water-soluble contrast, which confirmed intestinal perforation. However, the surgical exploration revealed perforation of the sigmoid colon at the posterior segment. The patient underwent antibiotic therapy, abdominal lavage, and colostomy, immediately. She was discharged in good condition approximately 14-days later.

**Conclusion:**

To our knowledge, this is the first report of sigmoid colon perforation in a term neonate following COVID-19.

## Background

Current knowledge regarding the impact of SARS-CoV-2 infection on both pregnant women and neonates has been linked with cases of maternal death, stillbirth, neonatal death, and preterm birth. In addition, the risk of miscarriage, the worse maternal outcomes, vertical transmission, and early-onset symptomatic neonatal SARS-CoV-2 infection are being raised. In addition, among COVID-19 patients, pregnant women seem to have worse outcomes than non-pregnant women [1]. Similarly, affected neonates have more severe illnesses than older children sub-populations [2]. Nevertheless, current data show that most infected newborns are in the spectrum of asymptomatic to mild symptomatic cases [3].

Although neonates may show more severe illness manifestations [4], it is rarely reported that infected neonates with COVID-19 present with intestinal perforation (IP) [5, 6].

The present study reports the rare presentation of early-onset sigmoid colon perforation in a term newborn with a positive SARS-CoV-2 RT-PCR test. The sigmoid colon is the terminal portion of the large intestine before reaching the rectum. Its location is usually in the pelvis. However, the perforation in this area is rare, and only a few cases of IP fall in full-term newborns out [7]. The presence of both of these two issues has made the recent report unique and interesting.

## Case presentation

A 2550 g term female newborn admitted to the Neonatal Intensive Care Unit (NICU) due to nasal flaring, tachypnea, and mild subcostal retraction. She born at 38 weeks of gestation and had a normal Apgar score in the first and fifth minutes. She born by cesarean section to a 21-year-old primigravida woman. In the absence of a history of previous illnesses, the mother had a history of new-onset moderate COVID-19 infection from 10-days before delivery, which led to her hospitalization. She had fever, cough, and reduced oxygen percentage to 90% at this period. Nevertheless, her disease was managed with oxygen and supportive care without antiviral therapeutic agents. On physical examination, the neonate had mild respiratory distress and the heart rate, respiratory rate, and blood pressure were 137/min, 62/min, and 70/50 mmHg, respectively. Oxygen saturation was 94% on oxygen, and the core temperature was 37 °C. Sepsis screening including blood culture, chest X-Ray, and all related lab tests (Table 1) was performed. The chest X-Ray was normal with no evidence of over or under aeration, prominent perihilar interstitial markings, opacification of the lung parenchyma, air-bronchogram, and pleural effusion. In addition, she evaluated for SARS-CoV-2 by RT-PCR, using the current pishtazteb kits, according to the manufacturer’s protocol, with a positive result [8]. Empirical antibiotics (Ampicillin and Amikacins) started to treat possible early sepsis, and intravenous fluid of dextrose 10% in the water began as well.

**Table 1:** The results of laboratory investigations of the neonate (on day 1, except for the last three which were done later)

Laboratory Parameter	Normal value	Early Results
White Blood Cell (WBC) count	4.5–10.0 × 10^3^/UL	8.5 × 10^3^
Red Blood Cell (RBC) count	4.2–5.4 × 10^6^/UL	5.14 × 10^6^
Hemoglobin	12.0–16 g/dL	14.5
Mean Corpuscular volume (MCV)	110 ± 15 fL	93.4
Platelet count	150–450 × 10^9^/UL	271 × 10^9^
C-reactive Protein (CRP)	< 5 mg/L	104
Urea	15–45 mg/dL	29
Creatinine	0.5–0.9 mg/dL	0.7
Bili. Total	< 2.0 mg/dL	2.6
Bili. Direct	< 0.5 mg/dL	1.1
Serum Albumin	> 4.0 g/dL	4.0
Blood glucose	> 45 mg/dL	76
Blood culture	No growth	Negative
Nasal RT-PCR	Negative	Positive
PH	7.35–7.45	7.48
HCO3	22–28 meq/L	18.2
PCO2	35–45 mmHg	24.2
Urine CMV PCR^a^	Negative	Negative
Intraperitoneal fluid analysis^b^	-Appearance: clear -Protein< 3 g/dL (0.3–0.4) -Albumin: Low -Serum-ascites albumin gradient (SAAG) > 1.1 g/Dl is considered transudate. -Cell count: Few (< 80/μL), Lymphocyte dominant (85%) - Glucose: Equal to the glucose level in the blood -Fluid Lactate dehydrogenase (LDH)/Serum LDH < 0.6 (transudate).	- cloudy, contains feces -Protein:3.5 g/dL -Albumin:3.0 g/dL -SAAG< 1.1 -WBC: 480 (PMN: 85%) -Glucose:42 mg/dL -Fluid LDH/serum LDH: NA^c^
Intraperitoneal fluid culture	No growth	Poly-microbial (Gram positive, gram negative, and anaerobic agents)

^a^On day 14

^b^On day 4

^c^*NA* Not assessed

The patients’ respiratory distress decreased during the second day, resulting once breastfeeding without tolerance. While she passed meconium and did not vomit during the first 48 hours of her life, she developed abdominal distention on day 3.

**Fig. 1:**
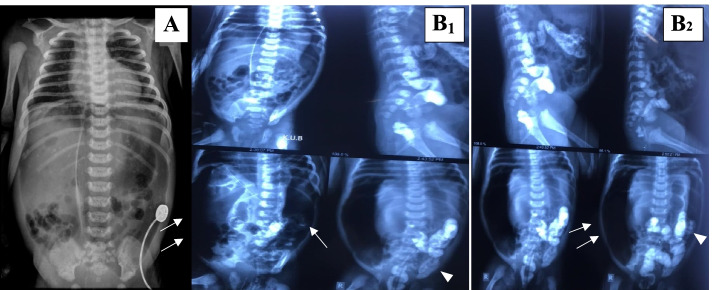
**A** Plain radiograph showing pneumoperitoneum. **B**_1_–_2_: water-soluble contrast enema, which indicated leakage of contrast (Arrowheads). Note the extensive pneumoperitoneum in the margins of the image (Arrows)

Surgical consultation was requested and a thoraco-abdominal X-Ray was performed which showed evidence of pneumoperitoneum (Fig. 1 A). Subsequently, antibiotics were switched to cefotaxime (100 mg/kg/day, divided in two doses), vancomycin (20 mg/kg/day, divided in two doses), and metronidazole (15 mg/kg stat, and then 7.7 mg/kg/day). The patient underwent an enema with a water-soluble contrast agent, which confirmed the diagnosis of intestinal perforation (Fig. 1 B_1_–_2_). However, the surgical exploration was performed on day 4 by a highly experienced pediatric surgeon. After washing the abdominal cavity which was contained a lot of digestive secretions and feces, the perforation site was found with difficulty in the posterior part of the sigmoid colon. The pediatric surgeon noticed this local perforation by passing normal saline through the gastric tract and seeing it exit at the sigmoid perforation site. A colostomy after resection was performed and the abdominal cavity was washed again. Two days after the colostomy, the patients’ general condition improved and abdominal distention reduced. On the sixth day of birth, low-volume breast milk was started until was fully fed. Finally, in the absence of pathological evidence of Hirschsprung disease, the patient was discharged in good condition approximately 14-days after abdominal lavage and colostomy. At this time the urine investigation for Cytomegalovirus (CMV) by PCR was performed with negative results**.**

## Discussion and conclusions

Although in preterm newborns, pneumoperitoneum is mostly due to necrotizing enterocolitis (NEC), in full-term infants the etiologic factors are different. In term neonates, risk factors include asphyxia, congenital heart disease, congenital hypothyroidism, intrauterine growth retardation, polycythemia, blood exchange, umbilical artery catheterization, and to a less extent NEC. However, a clear etiological factor cannot be determined in colonic IP, a situation in which there is a focal intestinal perforation (FIP) unrelated to the NEC. Sometimes, it is difficult to distinguish between these two conditions [9].

Historically, regardless of the route of viral transmission, cytomegalovirus has been implicated in such conditions [10]. Similarly, Daley et al. reported a newborn with disseminated herpes simplex virus infection associated with hematochezia and late sigmoid colon perforation [11]. Kora kaki et al. reported a case of a spontaneous, linear intestinal perforation in the transverse colon with urinary tract infection. In this case, none of the factors, which have previously been associated with IP, could be implicated. They suggested that FIP is possibly the result of infection [7].

The etiology and pathogenesis of FIP are still not clear. While hypoxia may lead to regional hypo perfusion and ischemia resulting in FIP [12], neither condition associated with perinatal asphyxia, nor any accompanying symptoms such as heart, kidney, or brain involvement, was matched with this condition.

In this case, the patient was born to a mother with a SARS-CoV-2 infection. Both of them had positive SARS-COV-2 RT-PCR results. Similarly, Harahap et al. reported a premature twin FIP of ileum that was born from a SARS-CoV-2 positive mother, albeit with negative SARS-COV-2 RT-PCR in the neonate [5]. Bindi et al. presented the case of a neonate that underwent abdominal surgery for IP on Meckel’s diverticulum with positive SARS-CoV-2 infection. The neonate was a three-day-old male infant that was transferred to the Salesi Hospital, Italy, with a history of feeding intolerance, biliary gastric stagnation, and abdominal distension. Although the pharyngeal testing of the neonate for SARS-CoV-2 was positive on the second postoperative day, the maternal swab result was negative at this time [6].

As far as we know, our case is the third IP and the first sigmoid perforation report among neonatal patients in the context of COVID-19. In contrast to the previous reports of FIP by Harahap et al., and Bindi et al., where both mother and neonate were not infected with SARS-CoV-2, our case is the first report of sigmoid colon perforation in a term neonate with SARS CoV-2 infection.

The optimal surgical management of IP is still controversial. An exploratory laparotomy by resecting the bowl and primary peritoneal drainage are two current approaches, with no difference in outcome in one prospective randomized trial study [13]. We performed peritoneal drainage and prepared a colostomy with acceptable results. Altogether, early diagnosis, immediate surgery, and appropriate postoperative care were the crucial determinant of the short-term prognosis of this patient.

To our knowledge, this is the first report of sigmoid colon perforation in a term neonate with a positive SARS-CoV-2 test, while the CMV test was negative and there were no other possibilities. In the absence of NEC and existing risk factors for the occurrence of spontaneous IP, the presence of SARS-CoV-2 can be considered as predisposing factors.

## Data Availability

The datasets used and/or analyzed during the current study are available from the corresponding author on reasonable request.

## Abbreviations

COVID-19: Coronavirus disease-2019
IP: Intestinal perforation
ICU: Neonatal intensive care unit
NEC: Necrotizing enterocolitis
FIP: Focal intestinal perforation
